# Recent Progress in the Understanding and Engineering of Coenzyme B_12_-Dependent Glycerol Dehydratase

**DOI:** 10.3389/fbioe.2020.500867

**Published:** 2020-11-05

**Authors:** Abdul Nasir, Somasundar Ashok, Jeung Yeop Shim, Sunghoon Park, Tae Hyeon Yoo

**Affiliations:** ^1^Department of Molecular Science and Technology, Ajou University, Suwon, South Korea; ^2^Bio R&D Center, Noroo Holdings Co., Ltd., Suwon, South Korea; ^3^School of Energy and Chemical Engineering, Ulsan National Institute of Science and Technology (UNIST), Ulsan, South Korea; ^4^Department of Applied Chemistry and Biological Engineering, Ajou University, Suwon, South Korea

**Keywords:** glycerol dehydratase, coenzyme B_12_, glycerol, inactivation, reactivase, enzyme engineering

## Abstract

Coenzyme B_12_-dependent glycerol dehydratase (GDHt) catalyzes the dehydration reaction of glycerol in the presence of adenosylcobalamin to yield 3-hydroxypropanal (3-HPA), which can be converted biologically to versatile platform chemicals such as 1,3-propanediol and 3-hydroxypropionic acid. Owing to the increased demand for biofuels, developing biological processes based on glycerol, which is a byproduct of biodiesel production, has attracted considerable attention recently. In this review, we will provide updates on the current understanding of the catalytic mechanism and structure of coenzyme B_12_-dependent GDHt, and then summarize the results of engineering attempts, with perspectives on future directions in its engineering.

## Introduction

Glycerol dehydratase (GDHt; EC 4.2.1.30) catalyzes the dehydration reaction of glycerol yielding 3-hydroxypropanal (3-HPA) that can be biologically converted into 1,3-propanediol (1,3-PD) or 3-hydroxypropionic acid (3-HP) by additionally expressing 1,3-propanediol dehydrogenase or aldehyde dehydrogenase, respectively, in microorganisms (Pawelkiewicz, 1965; Schneider et al., 1970; Stroinski et al., 1974; Toraya, 2000a; Huang et al., 2002; Liu et al., 2016; Park et al., 2017). 1,3-PD is used as a monomer for synthesizing polyethers, polyurethanes, and polyesters (Koutinas et al., 2014; Garlapati et al., 2016; Liu et al., 2016). 3-HP is a versatile platform chemical that can be converted into acrylic acid, acrylonitrile, and malonic acid (Valdehuesa et al., 2013; Chen and Liu, 2016; Kalantari et al., 2017). In addition, 3-HPA has an inhibitory effect on the growth of a wide variety of bacteria and therefore, prevents food spoilage, making it a suitable food preservative (Vollenweider and Lacroix, 2004). In particular, owing to the increase in the demand for biofuels, biological processes based on glycerol, a byproduct of biodiesel production, have been a recent focus of research (Da Silva et al., 2009; Ganesh et al., 2012; Cremonez et al., 2015; Ferrero et al., 2015). Besides glycerol, GDHt is also involved in the dehydration reaction of 1,2-propanediol to produce propanal, and the dehydration of 2,3-butanediol to yield butanone (Toraya et al., 1976; Chen et al., 2015).

Glycerol dehydratase are categorized on the basis of their reliance on coenzyme B_12_ (adenosylcobalamin (AdoCbl): coenzyme B_12_-independent GDHt and coenzyme B_12_-dependent GDHt (Yamanishi et al., 2002; Liao et al., 2003a; O’Brien et al., 2004). They share low sequence homology and are structurally very distinct despite the surprisingly similar architecture of the substrate-binding pockets (Liu et al., 2010; Martins-Pinheiro et al., 2016). Only coenzyme B_12_-independent GDHt from *Clostridium butyricum* has been experimentally characterized (O’Brien et al., 2004). The coenzyme B_12_-independent GDHt is extremely sensitive to oxygen and requires strict anaerobic conditions for its activity (Raynaud et al., 2003). However, most industrial microorganisms are cultured in the presence of oxygen, and the enzyme has rarely been used for the production of biochemicals so far. On the other hand, coenzyme B_12_-dependent GDHts are relatively resistant to aerobic conditions (Jiang et al., 2016), and have been utilized for bioconversion of glycerol (Liu et al., 2016). However, the complex cofactor, coenzyme B_12_, often undergoes chemical modifications during the reaction, resulting in catalytically inactive forms, and needs to be added in media for maintaining the enzyme activity. The dehydration reaction catalyzed by coenzyme B_12_-dependent GDHt has been reported as the rate-limiting step for the bioconversion of glycerol into 1,3-PD or 3-HP (Ahrens et al., 1998; Yuanyuan et al., 2004).

Coenzyme B_12_-dependent GDHt and its applications have previously been reviewed in several papers (Jiang et al., 2016; Liu et al., 2016; Jers et al., 2019). With increasing interest in the enzyme, studies on its biochemical features and engineering of the biocatalyst have recently been reported. In this review, we will provide updates on the current understanding of the structure and catalytic mechanism of B_12_-dependent GDHt, and in particular, describe its catalytic mechanism obtained through computational studies. To date, only limited attempts have been made to engineer coenzyme B_12_-dependent GDHt probably because of its multimeric structure and complicated reaction mechanism involving radicals. We summarize the findings from site-directed mutagenesis studies as well as recently reported engineering attempts. We also provide perspectives on the future directions in engineering coenzyme B_12_-dependent GDHt.

## Dehydration Reaction Catalyzed by Coenzyme B_12_-Dependent GDHt

**FIGURE 1:**
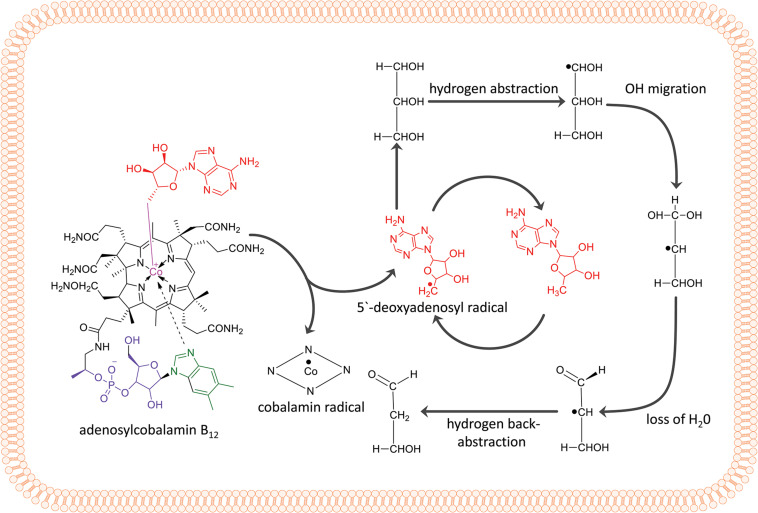
Adenylcobalamin B_12_ and the essential features of the dehydration reaction by coenzyme B_12_-dependent GDHt, including hydrogen abstraction, OH migration, loss of H_2_O, and hydrogen back-abstraction.

The overall reaction of GDHt is shown in Figure 1. The binding of a substrate such as glycerol induces conformational changes in the enzyme, which lengthens the bond between the Co atom and the adenosyl moiety of AdoCbl from 1.95–2.2 Å to 2.5 Å, followed by breakage of the C-Co bond (Toraya et al., 1977; Mancia et al., 1996; Shibata et al., 1999; Liao et al., 2003a). The adenosyl radical rotates alongside its glycosidic bond and abstracts the hydrogen atom from the substrate, resulting in the formation of a substrate radical (Frey, 1990, 2001; Mancia and Evans, 1998; Frey and Reed, 2000). Next, the OH group in the second carbon migrates to the terminal carbon, and a new radical is formed at the second carbon. The potassium ion present at the active site of the enzyme plays an important role in the OH migration during the catalysis (Shibata et al., 1999; Kamachi et al., 2007). The resulting 1,1-diol is unstable and is readily converted into an aldehyde group by releasing H_2_O. Next, the hydrogen atom is abstracted back to the substrate radical from 5′-deoxyadenosine, resulting in an aldehyde product and adenosyl radical.

## The Structure of Coenzyme B_12_-Dependent GDHt

**FIGURE 2:**
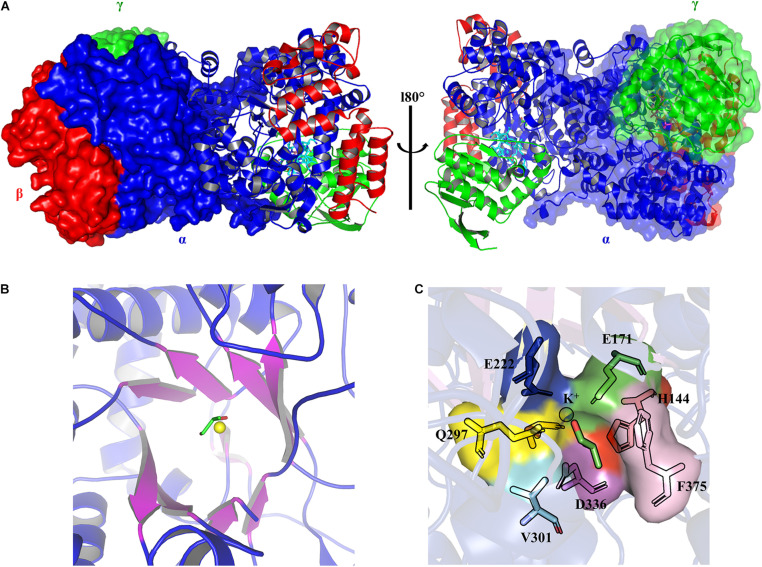
The three-dimensional structure models of *Kp*GDHt (PDB ID 1IWP). **(A)** The dimeric form of *Kp*GDHt. The α-, β-, and γ-subunits are shown in blue, red, and green color, respectively. The structures are shown by the ribbon or surface representation. **(B)** A topological diagram of the TIM barrel. The barrel β-sheets are shown in purple color. The substrate (shown by the stick model) and the K^+^ ion (yellow sphere) are present at the active site. **(C)** A zoom-in view of the active site residues interacting with the substrate and the K^+^ ion. The figures were created using PyMOL.

The crystal structures of the coenzyme B_12_-dependent GDHt from *Klebsiella pneumoniae* (*Kp*GDHt) in the presence or absence of a substrate have been reported (Yamanishi et al., 2002; Liao et al., 2003a). *Kp*GDHt exists as a dimer of αβγ-heterotrimer, (αβγ)_2_, and the dimerization is induced by the interaction of two α-subunits (Figure 2A). The β- and γ-subunits are separately bound to the α-subunit. AdoCbl resides between the α- and β-subunits of each αβγ-heterotrimer.

The α-subunit possesses the triosephosphate isomerase barrel (TIM) structure, where the substrate and the essential cofactor K^+^ are bound (Figure 2B). In the absence of a substrate, the potassium ion is hexacoordinated with amino acids at the active site and a water molecule (Liao et al., 2003a). The interaction is specific, and the K^+^ ion is unlikely to be exchanged with other monovalent cations such as ${\text{NH}}_{4}^{+}$ (Shibata et al., 1999). Binding the substrate breaks the bond with water, and the K^+^ ion is instead heptacoordinated with two OH groups of the substrate and five oxygen atoms from residues at the active site (Glu^α^ 171, Gln^α^ 142, Glu^α^ 222, Gln^α^ 297, and Ser^α^ 363) (Figure 2C; Yamanishi et al., 2002). Structural changes take place during the dissociation of the product from the enzyme, which leads to the positioning of the cofactor in its apo conformation (Yamanishi et al., 2002; Liao et al., 2003a; Toraya, 2003, 2014).

**FIGURE 3:**
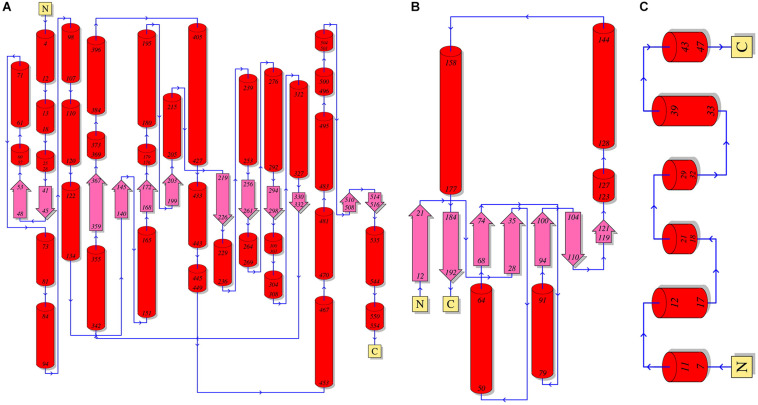
A schematic diagram of the secondary structures of the α-**(A)**, β-**(B)**, and γ-**(C)** subunits of coenzyme B_12_-dependent GDHt. The α-helices are shown as red cylinders and the β-strands as turquoise arrows.

A crack formed between the 10^th^ and 11^th^ β strands of the α-subunit has been assumed to be the path of substrate entry to the active site (Figure 3; Shibata et al., 1999). Three Asn and three Gln residues located around the crack possibly facilitate the entry of neutral hydrophilic compounds into the active site. The β-subunit forms the binding pocket for AdoCbl with the α-subunit, and the Rossmann fold-like structure in the central part of the β-subunit plays an important role in the interaction with the lower axial ligand of cobalamin (Liu et al., 2016). The γ-subunit is located far from the active site of the α-subunit and AdoCbl, and its role has been assumed to support the barrel structure of the α-subunit and the overall structure of coenzyme B_12_-dependent GDHt (Toraya, 2000a).

**FIGURE 4:**
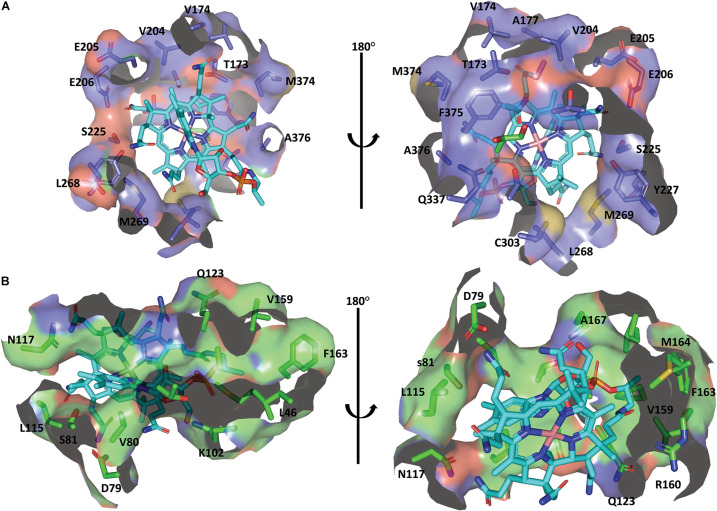
The binding mode of cobalamin in the *Kp*GDHt active site. **(A)** Residues of the α-subunit interacting with the cobalamin molecule. **(B)** Residues of the β-subunit interacting with the cobalamin molecule. The B_12_ molecule is shown by the cyan stick model. The figures were created using PyMOL.

Cobalamin derivatives are one of the most complex organic cofactors in nature. AdoCbl, the active form of cobalamin for coenzyme B_12_-dependent GDHt, resides in the pocket formed at the interface of the α- and β-subunits (Banerjee and Ragsdale, 2003; Maddock et al., 2015). Cobalamin has a complex structure consisting of cyclic tetrapyrroles, called corrin ring, with the cobalt atom at its center. The ring carries one nucleotide-derived tail comprising the dimethylbenzimidazole (DBI) group in addition to four propionamide, three acetamide, and eight methyl groups (Abeles and Dolphin, 1976; Banerjee, 1999; Figure 1). These peripheral groups interact with the residues in the α/β interface, which maintains the cofactor in a proper position for the radical-based catalysis (Figure 4; Shibata et al., 2018). The crystal structure of *Kp*GDHt reported by Yamanishi et al. has cyanocobalamin, an analog of AdoCbl (Rétey, 1990; Yamanishi et al., 2002). The cyanocobalamin-*Kp*GDHt complex structure provides information regarding how the peripheral groups of the corrin ring interact with the amino acid residues of the binding pocket (Shibata et al., 1999; Toraya, 2000a, 2014). Recently, the same group illustrated the complex structure of coenzyme B_12_-dependent diol dehydratase (DDHt) with AdoCbl (Shibata et al., 2018); coenzyme B_12_-dependent DDHt has nearly the same structure and catalytic mechanism as coenzyme B_12_-dependent GDHt (Toraya, 1994). This is the first complex structure including AdoCbl that shows how the native cofactor interacts with the enzyme. The adenosyl group of AdoCbl interacts with Thr^α^ 172 and Ser^α^ 224 whereas the ribose moiety is stabilized by two hydrogen bonds through the acetamide group of corrin and Ser^α^ 224. The substrate-free and substrate-bound structures exhibited the outward and inward shifts of the α-acetamide group, which was suggested to be linked to the opening and closing of a plausible channel for the passage of substrate and product (Shibata et al., 2018).

## Inactivation and Reactivation of Coenzyme B_12_-Dependent GDHt

**FIGURE 5:**
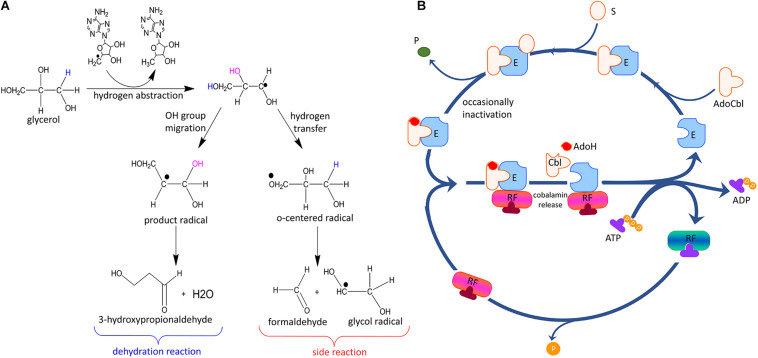
General mechanism of inactivation and reactivation of coenzyme B_12_-dependent GDHt. **(A)** Side reactions leading to the inactivation of coenzyme B_12_-dependent GDHt. **(B)** The mechanism of reactivation via cofactor exchange by GDHt reactivase. E: apoenzyme; AdoCbl: adenosylcobalamin; S: substrate; P: product; RF: reactivase; AdoH: 5′-deoxyadenosine; Cbl: cobalamin or damaged cofactor (Modified with permission from Bilicì et al., 2019).

Studies on coenzyme B_12_-dependent GDHts have shown reaction inactivation by glycerol (substrate-bound) or oxygen (apo-form), both of which are involved in the failure to regenerate AdoCbl in the catalytic cycle, yielding a tightly bound catalytically incompetent cobalamin at the active site of the enzyme (Figure 5A). The latter inactivation, known as physiological inactivation, is due to the cleavage of the partially activated Co-C bond via binding of oxygen to the bond, whereas in the former case, known as mechanism-based inactivation, irreversible homolysis occurs during the glycerol dehydration reaction (Toraya et al., 1976; Bachovchin et al., 1977; Toraya and Abeles, 1980; Toraya, 2000a; Tobimatsu et al., 2000; Seifert et al., 2001; Yamanishi et al., 2012).

**FIGURE 6:**
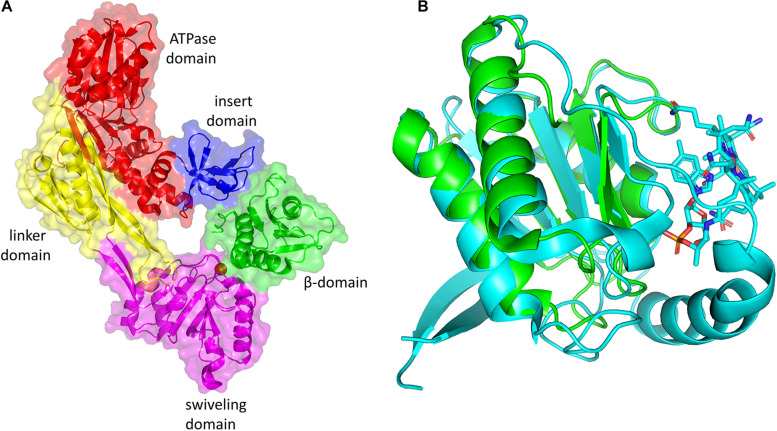
**(A)** Three-dimensional structure of GDHt reactivase αβ heterodimer (PDB ID: 1NBW). The ATPase, linker, swiveling, and insert domains of the α-subunit are colored red, yellow, magenta, and blue, respectively. The β- subunit is colored green. **(B)** Superimposition of the β-subunit of GDHt (cyan) and the β-subunit of GDHt reactivase (green). The B_12_ molecule is shown by the stick model. The figures were created using PyMOL.

The inactive cobalamin can be replaced with catalytically competent cobalamin by GDHt reactivase (Figure 5B) (Honda et al., 1980; Mori and Toraya, 1999). The structure of GDHt reactivase represents two αβ-heterodimers along with a hexacoordinated Mg^2+^ ion bound at the interface of the α- and β-subunits (Liao et al., 2003b). The α-subunit of GDHt reactivase contains four domains: the ATPase domain resembling those of molecular chaperons GroEL and Hsp70, the insert domain, the linker domain, and the swiveling domain (Figure 6A). Interestingly, the structure of the β-subunit of GDHt reactivase is similar to that of the β-subunit of GDHt (Figure 6B). The β-subunit swap hypothesis was proposed as a reactivation mechanism because of the structural features (Bennett et al., 1995; Liao et al., 2003b; Shibata et al., 2005). ATP hydrolysis by GDHt reactivase destabilizes the structure of the α-subunit, which facilitates its β-subunit dissociation from the swiveling domain of the α-subunit. Owing to their structural similarity, the β-subunit of GDHt reactivase binds to the α-subunit of GDHt replacing its β-subunit, during which the inactivated coenzyme B_12_ is released from GDHt (Yamanishi et al., 2002).

## Molecular Understanding of the Dehydration Reaction

**FIGURE 7:**
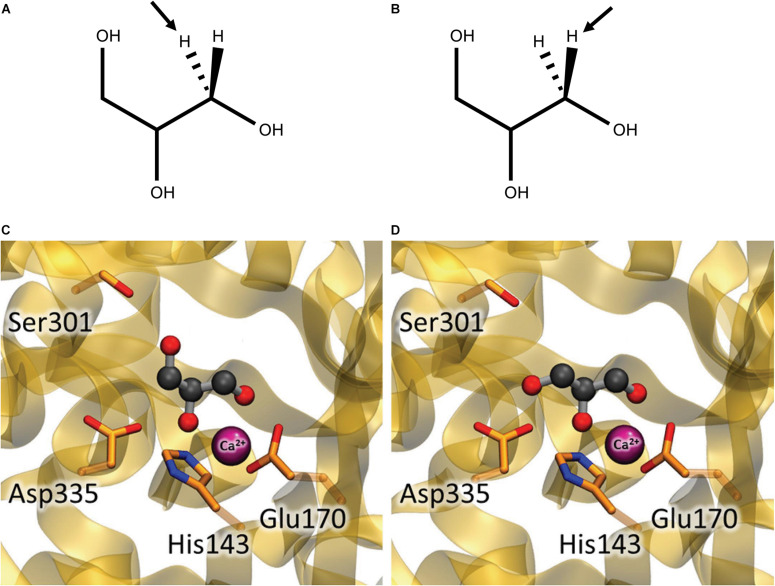
Glycerol in the pro-*S* **(A,C)** and pro-*R* **(B,D)** conformations at the active site of coenzyme B_12_-dependent DDHt. **(A,B)** Hydrogen abstracted in the first catalytic step are shown by arrows. **(C,D)** The C3-OH group of glycerol is oriented toward Ser301 in the pro-*S* conformation while toward Asp335 in the pro-*R* conformation (Modified with permission from Bilicì et al., 2019).

Coenzyme B_12_-dependent GDHt and coenzyme B_12_-dependent DDHt are isofunctional enzymes; they have the same catalytic mechanisms and are very structurally similar with a slight difference in substrate specificities. These extreme similarities suggest that these two enzymes possibly evolved from a common ancestor (Poznanskaja et al., 1979; Toraya, 1994, 1999, 2000b; Yamanishi et al., 2002; Liao et al., 2003a; Liu et al., 2010). Thus, catalytic mechanisms are discussed based on previous studies on DDHt and GDHt hereinafter. In a pioneering study on coenzyme B_12_-dependent DDHt from *K. pneumoniae* (*Kp*DDHt), Bachovchin et al. reported two binding modes of glycerol, the pro-*S* and pro-*R* conformations, depending on the position of the abstracted hydrogen (Figures 7A,B; Bachovchin et al., 1977). The dehydration reaction occurred dominantly when the substrate was bound in the pro-*R* conformation, whereas the inactivation reaction was preferable in the pro-*S* conformation. Doitomi et al. (2014) analyzed the three steps of substrate transformation (hydrogen abstraction, OH migration, and hydrogen re-abstraction) for both the pro-*R* and pro-*S* conformations using quantum mechanics/molecular mechanics methods. The C3-OH group in the pro-*S* conformation was oriented toward Ser301 of the α-subunit, and the hydrogen bond between them was suggested to increase the activation energy for the migration of the C2-OH group. Therefore, inactivation could take place prior to hydrogen recombination. In another computational study, Biliæ et al. also reported the interaction of the two conformations of glycerol at the active site of *Kp*DDHt with respect to the orientation of the C3-OH group. The OH group in the pro-*S* conformation was oriented toward Ser301 of the α-subunit, and that in the pro-*R* conformation was oriented toward Asp335 (Figures 7C,D; Bilicì et al., 2019). An attempt was made to introduce mutations into coenzyme B_12_-dependent DDHt to favor its interaction with the pro-*R* conformation of glycerol (Yamanishi et al., 2012), which will be described later.

## Engineering of Coenzyme B_12_-Dependent GDHt

### Improvement in Catalytic Activity

One approach to engineering enzymes is to introduce mutations at their active sites when the structures are available, in particular for the residues to interact with substrates (Yagonia et al., 2015; Lee et al., 2019). In addition, understanding the catalytic mechanism may provide useful insights into how a particular amino acid residue functions in enzymatic reactions. A study was reported on site-directed mutagenesis of the residues at the active site of coenzyme B_12_-dependent DDHt from *K. oxytoca* (*Ko*DDHt) (Kawata et al., 2006). Substitution into Ala was made for Gln^α^ 141, Gln^α^ 296, Ser^α^ 362, His^α^ 143, Glu^α^ 170, and Glu^α^ 221; Glu^α^ 170 was further mutated to Asp, Gln, or His. Asp^α^ 335 was mutated to Asp, Gln, and His, or Asn. All the mutations at Glu^α^ 170, Glu^α^ 221, and Asp^α^ 335 abolished the activity of *Ko*DDHt. Other variants also showed a decrease in activity compared to the wild-type (Table 1). These residues exhibited a lack of tolerance to mutagenesis, which demonstrates their important role in catalysis (Wilke et al., 2005). Yamanishi et al. identified two residues, Ser^α^ 301 and Gln^α^ 335, of *Ko*DDHt that play an important role in differentiating the two conformations of glycerol, the pro-*S* and pro-*R* conformations (Yamanishi et al., 2012); previous studies have demonstrated that glycerol in the pro-*S* conformation induces a mechanism-based inactivation (Toraya et al., 1976; Bachovchin et al., 1977). Substituting each of these residues with alanine showed a lower inactivation rate than the wild-type enzyme, but their enzyme activities decreased at the same time. This result suggests that the hydrogen bond interactions between the substrate 3-OH group and the active site residues have an important role in mechanism-based inactivation.

**TABLE 1:** Kinetic parameters of active site variants of coenzyme B_12_-dependent diol dehydratase.

Mutants	k_cat_ (s^–1^)		K_m_ (mM)		k_cat/_K_m_ (M^–1^. s^–1^) × 10^–6^		k_inact_ (min^–1^)		k_cat_/k_inact_ × 10^–4^		References
	1,2-PDO	Glycerol	1,2-PDO	Glycerol	1,2-PDO	Glycerol	1,2-PDO	Glycerol	1,2-PDO	Glycerol	
Wild-type	304	173	0.06	1.2	5.1	0.14	0.025	1.15	0.73	0.009	Yamanishi et al., 2012
S301A	247	90	0.38	1.2	0.65	0.08	0.052	0.22	0.28	0.025	
Q336A	109	30	0.57	1.7	0.19	0.02	0.058	0.081	0.11	0.022	
S301A/Q336A	98	67	2.4	0.84	0.04	0.08	0.36	0.66	0.016	0.006	
Q141A	150	–	0.04	–	1.5	–	0.26	–	3.5	–	Kawata et al., 2006
Q296A	170	–	0.1	–	0.017	–	0.56	–	1.8	–	
S362A	105	–	10	–	1.3	–	0.018	–	35	–	
H143A	5.1	–	0.08	–	–	–	1.8	–	0.017	–	
E170D	5.3	–	–	–	–	–	0.036	–	0.8	–	
E170Q	0.08	–	–	–	–	–	0.054	–	0.009	–	

Mutations that are far from the active site sometimes result in improvement in enzyme activity, in addition to other physical properties such as stability and solubility (Guan et al., 2004; Morley and Kazlauskas, 2005; Shukla et al., 2017). These variants are usually detected by screening the libraries generated via random mutagenesis. The substitutions can induce subtle changes at the active sites, possibly via the interaction network of the residues or structural dynamics, which has been reported to be related to enzyme activities (Mesecar et al., 1997; Whittle and Shanklin, 2001; Han and Shin, 2019). Qi and colleagues applied a directed evolution approach to a *Kp*GDHt library generated via a random mutagenesis method for improving catalytic activity and stability. The authors found two variants, Ile498Val of the α-subunit and Gln42Leu of the β-subunit, which demonstrated improved thermal and pH stability compared to the wild-type enzyme; the two positions were located far from the active site (Qi et al., 2009). Variants with a moderate increase in the catalytic efficiency toward glycerol were found in site-saturation libraries focusing on Ile^α^ 498 and Gln^β^ 42. Ile498Ala and Gln42Phe mutations increased the activity of glycerol by 1.8- and 8-fold, respectively (Qi et al., 2009). The two residues were then subjected to saturation mutagenesis. Interestingly, three variants, all of which have mutations at position 42 of the β-subunit, exhibited substantially improved catalytic efficiency (k_cat_/K_m_) toward both glycerol and 1,2-propanediol (Table 2). Another attempt was made to engineer *Kp*GDHt by introducing mutations using the PopMuSiC program (Kwasigroch et al., 2002), a computer-aided rational design program that predicts the thermodynamic stability changes caused by mutations (Qi et al., 2012). This study reported that the Tyr525Glu mutation in the α-subunit increased the catalytic activities of glycerol and 1,2-propanediol by 2- and 1.8-fold, respectively, whereas the α-Phe60Glu mutation showed opposite effects on the two substrates, increased the activity of 1,2-propanediol but decreased the activity of glycerol. These studies suggest that positions that are distant from the active site of the enzyme could be important targets for engineering.

**TABLE 2:** Kinetic parameters of coenzyme B_12_-dependent glycerol dehydratase variants.

Mutants	SA* (U^†^ mg^–1^)		K_m_ (mM)		Mutants	SA (U mg^–1^)		K_m_ (mM)	
	1,2-PDO	Glycerol	1,2-PDO	Glycerol	1,2-PDO	Glycerol	1,2-PDO	Glycerol	
^1^Wild-type	86	300	0.24	0.60	^1^Q42F	190	2500	0.19	2.61
^1^I498A	180	550	0.95	0.58	^1^Q42G	94	200	0.65	0.6
^1^I498C	99	180	0.24	0.60	^1^Q42H	88	920	0.55	0.66
^1^I498D	27	78	1.00	1.50	^1^Q42I	100	500	0.64	1.07
^1^I498M	130	310	0.012	0.50	^1^Q42K	43	730	0.27	2.80
^1^I498N	170	230	0.09	0.55	^1^Q42L	5	900	0.77	0.40
^1^I498P	49	120	1.90	0.90	^1^Q42M	190	440	0.24	1.18
^1^I498S	93	83	0.37	0.67	^1^Q42N	45	910	0.19	0.60
^1^I498T	83	66	3.30	0.52	^1^Q42P	77	1400	0.31	0.70
^1^I498V	120	310	0.17	0.59	^1^Q42R	26	110	0.14	0.44
^1^I498W	38	37	0.19	0.62	^1^Q42S	11	930	0.45	0.48
^1^Q42A	20	140	0.75	1.80	^1^Q42T	96	280	1.38	0.82
^1^Q42C	90	380	1.13	1.22	^1^Q42V	18	150	1.54	1.30
^1^Q42D	180	590	0.19	1.94	^1^Q42W	270	980	0.54	0.57
^1^Q42E	18	80	0.19	0.62	^1^Q42Y	22	270	0.19	1.03
^2^F60E	125	–	0.2	0.6	^2^Y525E	90	–	0.2	0.7

*SA = Specific activity – the number of enzyme units divided by the amount of enzyme; ^†^One unit (U) is the amount of enzyme that consumes 1 μmol substrate per minute; ^1^Data from Qi et al., 2012; ^2^Data from Qi et al., 2009.

### The Fusion of α- and β-Subunits

**FIGURE 8:**
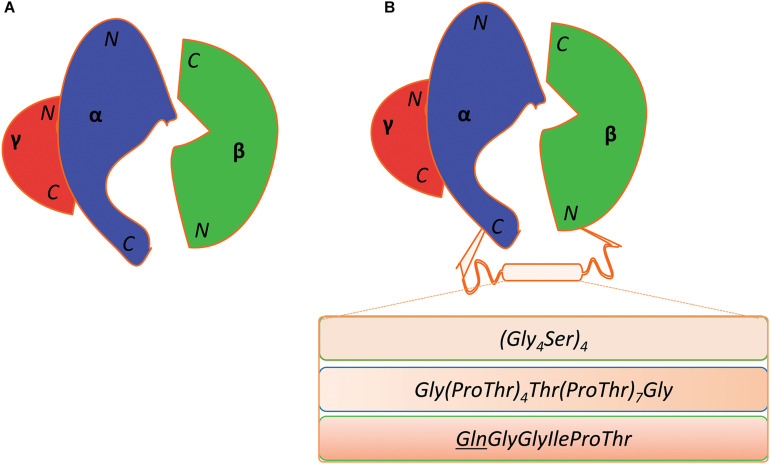
Fusion of the α- and β-subunit of coenzyme B_12_-dependent GDHt. **(A)** Diagrammatic representation of the wild-type coenzyme B_12_-dependent GDHt. Their N- and C-termini of the α-, β-, and γ-subunits are indicated. **(B)** The three different linkers are shown in the box between the C-terminus of the α-subunit and the N-terminus of the β-subunit. The mutation of the stop codon (TAA) of the α-subunit into a Gln codon (CAA) resulted in the third linker; the Gln position was underlined in the sequence.

The β-subunit of *Kp*GDHt is prone to dissociate from the enzyme complex during purification (Yamanishi et al., 2002). This problem could be circumvented through the fusion of the α- and β-subunits via a peptide linker; the C-terminus of the α-subunit is located close to the N-terminus of the β-subunit (Figure 8; Wang et al., 2009; Maddock et al., 2017). Wang et al. fused the α- and the β-subunits of *Kp*GDHt using a 20-residue linker of (Gly_4_Ser)_4_, and the engineered enzyme exhibited comparable catalytic activities (k_cat_/K_m_) to the wild-type enzyme (Wang et al., 2009). Maddock et al. adopted a linker of G(PT)_4_T(PT)_7_G from endoglucanase A of *Cellulomonas fimi*, and the enzyme unexpectedly showed a 20°C increase in the optimal temperature for the activity toward 1,2-propanediol (Maddock et al., 2017). In an attempt to engineer *Kp*GDHt to improve its resistance to inactivation, an interesting variant was isolated which has a mutation at the stop codon (TAA) of the α-subunit to CAA (Gln), resulting in the fusion of the α- and β-subunits (Gibson et al., 2013). The fused enzyme showed a slower inactivation rate than the wild-type enzyme in an assay using the cell lysate.

### Alternation of Substrate Specificity

*Kp*GDHt has a promiscuous activity to dehydrate 2,3-butanediol to butanone, as there is no known enzyme for the reaction (Zhang et al., 2014; Chen et al., 2015). Butanone is an industrial solvent, used in the manufacture of paints, wood coatings, adhesives, inks, and pharmaceuticals. Maddock et al. reported that the catalytic efficiency (k_cat_/K_m_) of *Kp*GDHt toward 2,3-butanediol was several hundred-fold lower than that toward 1,2-propanediol (Maddock et al., 2017). They applied strategies of combinatorial active site saturation and consensus-guided mutagenesis to improve the activity of *Kp*GDHt toward meso-2,3-butanediol. It had been hypothesized that starting from a more stable protein would increase the rate of success in the protein engineering based on the observation that stable proteins are more resistant to mutations (Gummadi, 2003; Bloom et al., 2006). Maddock et al. (2017) used a fused enzyme, in which the α- and β-subunits of *Kp*GDHt were linked via a Pro-rich linker showing much higher stability than the wild-type enzyme, as a template for generating libraries and found that a single point mutation (α-Thr200Ser) resulted in a four-fold increase in the catalytic efficiency of *Kp*GDHt toward meso-2,3-butanediol by screening over 5,500 variants.

## Perspectives on the Engineering of Coenzyme B_12_-Dependent GDHt

Coenzyme B_12_-dependent GDHt is the key enzyme in the biological conversion of glycerol into 1,3-PD or 3-HP, and it has been utilized in developing processes producing them. The enzyme, however, has a critical drawback of losing its activity resulting from the modification of AdoCbl, and its reactivation needs the action of GDHt reactivase involving the consumption of ATP. Supplementation of coenzyme B_12_ in media is necessary even for microorganisms naturally synthesizing the cofactor to attain high productivity. Therefore, engineering an enzyme that is resistant to inactivation should be an important research direction in applying coenzyme B_12_-dependent GDHts for industrial processes. Structural and computational studies have demonstrated that the conformation of glycerol plays a role in GDHt inactivation (Bachovchin et al., 1977; Bilicì et al., 2019). Mutations were introduced at the active site of coenzyme B_12_-dependent DDHt to favor its interaction with the pro-*R* conformation, and the variants showed slower inactivation rates than the wild-type enzyme even though their activities decreased (Yamanishi et al., 2012). The results suggest some possibilities to improve resistance to inactivation by engineering the substrate-binding site of coenzyme B_12_-dependent GDHts.

An engineered enzyme in which the α- and β-subunits were fused via a linker displayed a higher resistance to inactivation than the wild-type enzyme (Gibson et al., 2013). The result suggests that linking the two subunits is an alternative strategy to engineer the enzyme particularly for resisting inactivation. However, the result was demonstrated in an assay using cell lysate, and the fused enzyme needs further characterizations before a conclusion can be drawn. In particular, how fusion affects reactivation by GDHt reactivase remains unknown. Linkers to connect the two proteins can affect the properties of the fused proteins (Reddy Chichili et al., 2013; Morales-Luna et al., 2018; Rullán-Lind et al., 2018). A few linkers have been utilized so far, and only the case mentioned above was investigated with respect to inactivation. Systematic studies on the linkers to connect the two subunits can yield engineered variants more resistant to inactivation. The study by Liu and colleagues revealed that some bacterial species, such as *Mesorhizobium loti* and *Mycobacterium smegmatis*, express GDHt enzymes in the form of an αγ-heterodimer where the α-subunits have additional sequences homologous to the β-subunit of *Kp*GDHt (Liu et al., 2010). These enzymes would be candidates for application in the bioconversion of glycerol.

## Ethics Statement

This manuscript does not include any data from studies involving animal or human subjects.

## Author Contributions

All authors listed have made a substantial, direct and intellectual contribution to the work, and approved it for publication.

## Conflict of Interest

SA is employed by the Noroo Holdings Co., Ltd. The remaining authors declare that the research was conducted in the absence of any commercial or financial relationships that could be construed as a potential conflict of interest.
